# Natural Killer-Based Therapy: A Prospective Thought for Cancer Treatment Related to Diversified Drug Delivery Pathways

**DOI:** 10.3390/pharmaceutics16070939

**Published:** 2024-07-14

**Authors:** Jing Zang, Yijun Mei, Shiguo Zhu, Shaoping Yin, Nianping Feng, Tianyuan Ci, Yaqi Lyu

**Affiliations:** 1 School of Pharmacy, Shanghai University of Traditional Chinese Medicine, Shanghai 201203, China; zangjing97@163.com (J.Z.); npfeng@hotmail.com (N.F.); 2 State Key Laboratory of Natural Medicines, Department of Pharmaceutics, School of Pharmacy, China Pharmaceutical University, Nanjing 210009, China; yjmei@stu.cpu.edu.cn; 3 Department of Immunology and Pathogenic Biology, School of Integrative Medicine, Shanghai University of Traditional Chinese Medicine, Shanghai 201203, China; zhushiguo@shutcm.edu.cn; 4 School of Pharmacy, Jiangsu Provincial Engineering Research Center of Traditional Chinese Medicine External Medication Development and Application, Nanjing University of Chinese Medicine, Nanjing 210023, China; ysp0305@126.com

**Keywords:** drug delivery system, tumor immunity, exosomes, NK cell activation

## Abstract

Immunotherapy has been a research hotspot due to its low side effects, long-lasting efficacy, and wide anti-tumor spectrum. Recently, NK cell-based immunotherapy has gained broad attention for its unique immunological character of tumor identification and eradication and low risk of graft-versus-host disease and cytokine storm. With the cooperation of a drug delivery system (DDS), NK cells activate tumoricidal activity by adjusting the balance of the activating and inhibitory signals on their surface after drug-loaded DDS administration. Moreover, NK cells or NK-derived exosomes can also be applied as drug carriers for distinct modification to promote NK activation and exert anti-tumor effects. In this review, we first introduce the source and classification of NK cells and describe the common activating and inhibitory receptors on their surface. Then, we summarize the strategies for activating NK cells in vivo through various DDSs. Finally, the application prospects of NK cells in tumor immunotherapy are also discussed.

## 1. Introduction

Cancer has become an intractable problem affecting human health around the world; nevertheless, there has been no universal tumor treatment strategy up to now [1]. Surgical resection, chemotherapy, radiotherapy, and immunotherapy are generally the common methods for treating cancer in clinical practice, and surgical resection is still the most effective way for eradicating tumors in the early stage [2,3,4,5]. Unfortunately, due to the lack of surgical conditions and insufficient therapeutic effect, less than 25% of cancer patients can receive surgical resection in time worldwide [6,7]. Although chemotherapy and radiotherapy are also supposed to directly inhibit tumor progression besides surgery, both of them result in poor patient compliance and may cause serious adverse effects like cardio cytotoxicity, alopecia myelotoxicity, neurotoxicity, and nephrotoxicity [8,9,10,11]. Since the Nobel Prize in Physiology and Medicine was produced in the field of immunotherapy in 2018, immunotherapy has been attracting more and more attention among numerous therapies against various types of cancer owing to its enhanced anti-tumor efficacy, attenuated side effects, and long-term immunological memory for preventing tumor metastasis and recurrence [12,13,14,15]. Tumor cells can be eliminated during immunotherapy through the activation of the immune system, subsequently playing a long-term “surveillance” role in patients’ bodies [16].

Natural killer (NK) cells are a type of critical immunocytes for immunotherapy, which can directly recognize and kill tumor cells without prior stimulation of tumor antigens, and seldom cause side effects like inflammatory storm. Thus, NK cells are regarded as another hot research direction alongside T-cell therapy [17]. NK cells are the third largest type of lymphocytes other than T cells and B cells, which were first discovered in 1975 by Herberman and Kiessling [18,19,20]. NK cells mainly develop from lymphoid progenitors in bone marrow, while some secondary lymphoid organs, such as the tonsils, spleen, and lymph nodes, also show a close relation to the maturation of NK cells [21]. Mature NK cells belong to large granular lymphocytes in morphology, which mainly distribute in peripheral blood, lymph nodes, the spleen, and bone marrow, accounting for 5 to 15% of the lymphocytes in human peripheral blood [22]. There are many commonly used methods for identifying and characterizing NK cells. For example, the NK cells in human peripheral blood, tissues, and bone marrow could be determined by the lack of CD3 on their surface, but they express neural cell adhesion molecule (NCAM, also named as CD56), while the ones in circulatory system could be determined by the expression of natural cytotoxic triggering receptor 1 (NCR1, also named NKp46 or CD335) [23,24,25,26]. NK cells can be further divided into CD56^bright^CD16^dim−^ and CD56^dim^CD16^+^ subsets according to their maturation status. The less mature subset CD56^bright^CD16^dim−^ is the main producer of cytokines, while CD56^dim^CD16^+^ (the more mature subset) mainly exerts cytotoxic effects [27,28,29].

NK cells simultaneously possess cytotoxic and immunomodulatory characteristics without major histocompatibility complex (MHC) restriction, monitoring and inhibiting tumor growth in various ways [17,30]. NK cells regulate their functions (cytotoxicity, proliferation, and cytokine production) based on the stimulation balance between activating and inhibition receptors expressed on the surface of the cell membrane, subsequently activating the apoptotic pathway of tumor cells without prior sensitization [31]. Cytokine secretion is an important modality for NK cells to kill tumor cells. NK cells are the major source of distinct cytokines, producing tumor necrosis factor-α (TNF-α) and interferon-γ (IFN-γ) with tumoricidal properties [21,32]. TNF-α usually plays an essential role in tumor treatment, which can directly kill tumor cells without significant toxicity to normal cells. TNF-α shows its cytotoxicity, antiviral property and immune regulating functions by binding to the tumor necrosis factor receptor (TNFR) on target cells, enhancing the host’s immune function or acting with tumor vascular endothelial cells for vascular dysfunction [33,34,35]. IFN-γ is another critical cytokine during tumor therapy, which can induce tumor cell apoptosis through the IFN-γ/STAT1 pathway, and the activation of STAT1 also inhibits tumor growth and establishes a dormant state [36]. In addition, NK cells release cytotoxic substances like perforin and granzyme, eradicating tumor cells through an antibody-dependent cell-mediated cytotoxicity (ADCC) effect [37,38].

To enhance drug bioavailability and reduce side effects, drug delivery systems (DDSs) have been widely used in chemotherapy delivery. DDSs typically consist of two parts: drugs and their carriers. These systems can be categorized into two types: active targeting DDSs and passive targeting DDSs. The active targeting DDSs are usually modified with antibodies, proteins, nucleic acids, etc., which can bind to specific receptors or antigens at the target site, thus increasing the drug concentration at the target sites [39,40]. In contrast, passive targeting DDSs are taken up by the target site through mechanisms such as capillary interception, intracellular endocytosis, cell membrane fusion, adsorption, and enhanced osmotic retention [41,42]. Adopting DDSs can stimulate the activation of NK cells in vivo to exert their anti-tumor activity, while the NK cells can also be directly applied as tumor-targeted drug delivery carriers with the previous load of antibodies or DDS in vitro for enhanced tumor therapy. The anti-tumor property of NK cells can be activated by DDSs in various ways. For example, DDSs can enhance the activation signal of NK cells by stimulating the expression of NK-activating receptors or upregulating their ligands on tumor cells, or by blocking the expression of inhibitory receptors and their ligands [43,44,45]. In addition, there are a large number of modifiable groups on the surface of NK cells and NK-derived exosomes (NEOs), and the endocytosis ability possessed by NK cells also enables them to be directly applied as drug delivery carriers for loading DDSs and exerting anti-tumor effects [46,47]. In this review, we aimed to initially introduce both the stimulatory and inhibitory receptors expressed on the surface of NK cells, then describe the application of DDSs to activate NK cells via controlling the balance between stimulatory and inhibitory signals during tumor immunotherapy. Finally, we summarize the strategies of adopting NK cells and NEOs as drug delivery vehicles for anti-tumor therapy.

## 2. Receptors Expressed on NK Cells

**Figure 1:**
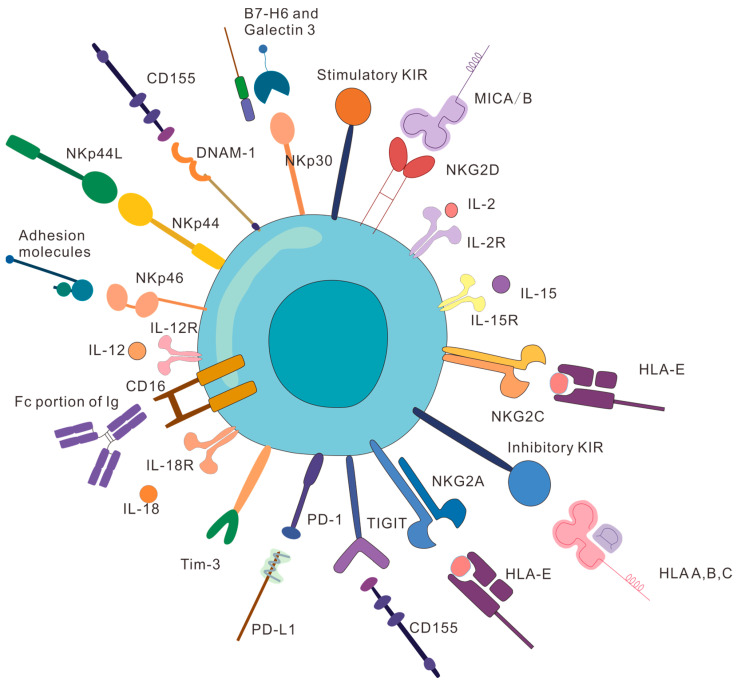
Schematic illustration of representative activating and inhibitory receptors and some ligands on the surface of NK cells.

Generally, the activation of T cells for tumor eradication can be achieved after their recognition of the antigens presented by MHC molecules on the surface of antigen-presenting cells (APCs) [48,49]. In comparison, the activation or inhibition of NK cells is determined by the balance between both stimulatory and inhibitory signals on their surface without MHC restriction [31,50]. When the activation signal is stronger than the inhibition one, the NK cells will be activated and later kill the target cells; otherwise, these NK cells will remain silent (Figure 1) [51]. As tumor cells generally lack or even do not express MHC-I to escape from being recognized by T cells, NK cells exhibit outstanding application prospects for tumor immunity [52]. NK cells can recognize tumor cells without high MHC-I expression, making up for the deficiency of T cells, and play an important role in cancer treatment [53].

### 2.1. Activating Receptors on NK Cells

There are numerous types of activating receptors expressed on the surface of NK cells, which are responsible for NK triggering when they are interacting with the specific ligands expressed under stress, viral infection, or transformed cells [17]. NKG2D is the most typical activating receptor on NK cells with the ability to recognize the structural analogs of MHC-I like ULBP and MICA/B, which can be upregulated through infection, stress, and tumor cells, and plays a critical role in processing tumor immune surveillance [54]. CD16 is another important activating receptor expressed by NK cells. The Fab segment of the IgG antibody binds to the epitope of virus-infected or tumor cells, while the Fc segment of the IgG antibody binds to the CD16 on the NK cell surface, inducing NK cells to directly kill target cells [55,56]. Cytotoxicity receptors (NCRs, including NKp46, NKp30, and NKp44) also play a core role in activating NK cells. NKp30 can bind to B7-H6 and BAG6 on tumor cells to trigger NK activation [57], and NKp46 is a 46 kDa glycoprotein composed of two C2-type extracellular Ig-like domains for NK activation, cytotoxic activity, and cytokine production by inducing protein tyrosine kinase-dependent signaling pathways [58]. Both NKp30 and NKp46 promote NK activation by interacting with ITAM-containing adapter proteins, while NKp44 activates NK cells by interacting with Dap12 [59,60].

In addition, NK cells possess distinct cytokine receptors like interleukin 2 receptor (IL-2R), interleukin 12 receptor (IL-12R), interleukin 15 receptor (IL-15R), interleukin 18 receptor (IL-18R), and interleukin 21 receptor (IL-21R) on their surface. Applying cytokines for promoting both the activation and the proliferation of NK cells is regarded as one of the most common strategies to enhance NK cells’ tumoricidal ability [61,62]. For example, interleukin 2 (IL-2) is the first cytokine for NK activation in clinical practice, which has been approved by the FDA for treating metastatic kidney cancer and melanoma [63]. Exogenous cytokine IL-2 not only induces continuous NK expansion and activation, but also enhances the maturation and persistence of isolated NK cells. IL-12 can stimulate the expression of chemokine (C-X-C motif) ligand 6 (CXCR6) and CD49a on NK cells in human peripheral blood, enhancing the expression of IFN-γ and NKG2C, but the use of IL-2 for cancer treatment may cause severe systemic toxicities [64]. Interleukin 15 (IL-15) amplifies the NK population and increases the expression of NK activating-receptors without inducing the expansion of Treg populations [65].

Other common NK-activating receptors include DNAM-1, NKG2C, NKG2E, NKG2H, killer cell immunoglobulin-like receptors (KIRs, including KIR-2DS and KIR-3DS), etc. [66,67]. After recognizing the ligands (CD155 and CD112), DNAM-1 not only promotes NK-mediated clearance of transformed and virus-infected cells, but also plays a key role in the expansion and maintenance of virus-specific memory NK cells [68]. In summary, upregulating the expression of NK-activating receptors or enhancing the binding to their ligands can facilitate NK activation.

### 2.2. Inhibitory Receptors on NK Cells

NK cells also express a variety of inhibitory receptors on their surface to counteract stimulatory signals produced from the activating receptors [69]. Due to the fact that the activation state of NK cells is determined by the balance between stimulating and inhibitory signals, reducing the binding between inhibitory receptors to their ligands or inhibiting the expression of inhibitory receptors or their ligands can also effectively stimulate the anti-tumor efficacy of NK cells. Natural Killer Group 2A (NKG2A), a member of the C-type lectin-like NKG2 receptor family, is an important inhibitory receptor expressed by NK cells, accounting for about 20~80% of all receptors on healthy NK cells, and prevents them from accidentally injuring normal cells [70,71,72]. Programmed death 1 (PD-1) is also a surface inhibitory receptor on NK cells [73]. PD-1 blockage can enhance the eradication of NK cells to multiple myeloma cells and improve the release of IFN-γ from NK cells [74]. T-cell immunoglobulin and mucin domain-containing protein-3 (Tim-3) is another type of inhibitory receptor on NK cells, and the activation of NK cells can be inhibited after binding phosphatidylserine (PS) with Tim-3 [75].

## 3. Drug Delivery System-Based NK Activation for Tumor Immunotherapy

Compared with T cells that need to be stimulated by tumor antigens presented by APC, the non-restricted killing of NK cells has attracted more and more attention in tumor immunotherapy. Nevertheless, some shortcomings of NK cells, such as insufficient invasion, biological activity, and immune surveillance, may diminish their tumor eradication ability to a certain extent. NK cells can be activated either by the balance of activating or inhibitory receptors on their surface, or by the action of ADCC to kill tumors. As is shown in Table 1, the most commonly adopted method for in vivo NK activation through DDS can be summarized as enhancing the activation signals and reducing the inhibitory signals of NK cells.

### 3.1. Ligand Expression Stimulation of Activating Receptors

Regulating the expression of activating receptors and their ligands through DDSs can destroy the transduction balance between stimulus and inhibition signals, overcome the immunosuppressive state, and activate the anti-tumor property of NK cells [44]. Shi et al. designed a core–shell-structured and Zn^2+^-doped Zn-CoFe_2_O_4_@Zn-MnFe_2_O_4_ superparamagnetic NP (ZCMF) to treat hepatoma [43]. ZCMF showed highly controllable magnetic hyperthermia performance and ensured synergistic anti-tumor effects with mild magnetothermal therapy (MHT). As the mononuclear phagocyte system in the liver tends to capture intravenous-injected magnetic NPs, ZCMF could be abundantly accumulated in the liver after administration. Through MHT treatment, ZCMF significantly increased the expression of UL16-binding proteins (ULBPs) on the surface of HepG2 cells (human hepatoma cells). ULBPs are ligands for NKG2D, and the upregulation of ULBPs stimulates the activation of NK cells. According to the experimental results, the growth of both xenograft and orthotopic liver tumors was almost completely eradicated by inducing in vivo NK-related anti-tumor immunity.

Huang et al. uniformly combined polymer-coated carbon nanodots into an ordered mesoporous silica framework to obtain CD@MSN [90]. CD@MSN was a type of biodegradable DDS for photothermal therapy. After CD@MSN treatment, the expression of activating receptors (NKG2D and NKp46) on NK cells obviously increased compared with a mesoporous silica NP (MSN) group. In addition, NK activation-related proteins, NKG2D ligands (including H60 and mrn41 proteins) on 4T1 cells (murine breast cancer cells), were also increased after NIR irradiation, hence stimulating the proliferation and activation of NK cells in vivo.

Another study has demonstrated that ruthenium (Ru)-based metal complexes possess outstanding tumor treatment efficacy and satisfy biosafety requirements. A Ru polypyridine complex (RuPOP) could regulate NK activation-related proteins on tumor cells to enhance the killing ability of NK cells against tumors. Based on this point, Xu et al. synthesized this DDS to enhance the expression of various ligands (MICA/B, ULBP1, ULBP2, ULBP4) for further NKG2D activation on MDA-MB-231 cells (human breast cancer cells). After therapy, the interaction between NK cells and tumor cells was strengthened [89].

### 3.2. Cytokine-Related Application

NK cells generally express distinct cytokine receptors (IL-21R, IL-2R, IL-12R, IL-15R, IL-18R, etc.) on their surface, and some pro-inflammatory cytokines like IL-2 and IL-15 can directly bind to their related receptors on NK cells for stimulation [96,97]. Activating NK cells through cytokines is one of the relatively mature methods to strengthen the anti-tumor characteristic of NK cells [62]. Cytokine-based NK stimulation has also been used in clinics or has reached the clinical trial phase, such as the application of Bempegaldesleukin (also named BEMPEG or NKTR-214, an IL-2 pathway agonist) for treating melanoma [98], and the short-term continuous infusion of IL-15 (NK expansion) for treating lymphoma [99].

**Figure 2:**
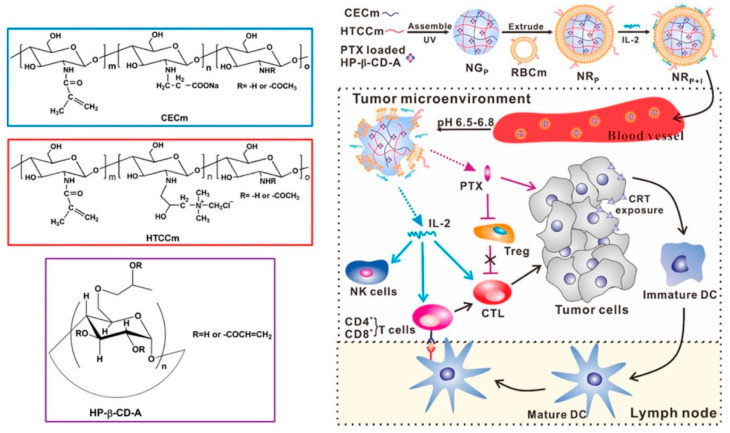
Schematic illustration of the preparation and functional mechanism of NR_P+I_. After accumulating in tumors through the EPR effect, NR_P+I_ was rapidly expanded in acidic TME and released PTX to induce CRT exposure on tumor cells. Subsequently, IL-2 was continuously released into the TME to activate CTL and NK cells. Picture reprinted from Ref. [78].

Direct cytokine application: Cytokines with NK activation effects can be directly loaded into DDSs for precise tumor delivery and further activate NK cells. IL-2 is an important cytokine regulating the survival, proliferation, and differentiation of activated NK cells, which can directly promote NK activation after administration. Zhang et al. from the Huazhong University of Science and Technology constructed a TME-sensitive red cell membrane-coated nanogel (NR) containing both paclitaxel and IL-2 (NR_P+I_) to treat tumors with the synergism of chemotherapy and immunotherapy (Figure 2) [78]. As IL-2 could promote the proliferation and activation of NK cells, the absolute amount of NK cells (CD3^−^NK1.1^+^) in murine melanoma tumors (B16-F10 cells) after treatment with NR_P+I_ was 1.6 and 1.5 times higher than PTX + IL-2 and NR_I_ groups, respectively. Zhang from Shandong University developed two types of micelles exhibiting both pH and redox sensitivity, namely IL-15-loaded micelles (IL-15-NPs) and Trp2/CpG-coloaded micelles (Trp2/CpG-NPs), which enhanced the tumoricidal effect of NK cells after interacting with IL-15-NPs, and promoted T-cell activation through DC antigen presentation and macrophage repolarization through Trp2/CpG-NPs, respectively [80]. The experimental results demonstrated that after treatment with IL-15-NPs, the infiltration of NK cells in B16-F10 tumors increased obviously, and the tumor inhibition rate reached 93.76%. Meanwhile, there is also an increased infiltration of both T cells and NK cells in tumors.

Adoption of cytokine-encoded plasmids or mRNAs: In addition to the direct delivery of cytokines, plasmids or mRNAs encoded with cytokines can also be loaded by DDSs for NK activation. Gao et al. prepared RDMCM NPs (RDMCM/pIL-12 + PD-1/PD-L1i) composing two copolymers (cRGD-PEG-PCL and MPEG-PCL-MPEG) and DOTAP for loading an IL-12-encoded plasmid (pIL-12) and PD-1/PD-L1 small-molecule inhibitor (PD-1/PD-L1i) for melanoma (B16-F10 cells) therapy [79]. After intravenous injection, the above dual drugs were accurately delivered to tumors through the targetability to integrin αvβ3 receptor, and the expressed IL-12 on tumor cells was upregulated through the cellular uptake of pIL-12. This phenomenon indicated that the amount of activated NK cells in the RDMCM/pIL-12 + PD-1/PD-L1i group was almost twice that of the control group. Dong et al. developed a type of lipid NP (DAL-LNPs) for encapsulating mRNAs encoding multiple cytokines (including IL-12, IL-27, and GM-CSF) for B16-F10 melanoma treatment [84]. IL-12 activated both T cells and NK cells through the Stat4 pathway, while IL-27 could activate these cells through the Stat1 and Stat3 pathways. After intratumoral injection, the enhanced intratumoral infiltration of NK cells was achieved.

Stimulation of cytokine secretion: Some DDSs can stimulate the body to secrete various cytokines for inducing NK activation. Perry et al. designed particle replication in nonwetting templates (PRINT) NPs as the nanoplatform to deliver CpG (potent Toll-like receptor TLR9 agonist) to mouse lungs via oral duct infusion [82]. The combination of both CpG and TLR9 initiated a series of innate and acquired immune responses, which activated DC cells and subsequently promoted the secretion of pro-inflammatory cytokines (namely IL-6, IL-12, etc.) for NK activation. The results demonstrated that local delivery of PRINT-CpG to the lungs effectively promoted substantial tumor regression and limited systemic toxicity in two murine orthotopic metastasis models (344SQ cells: lung adenocarcinoma cells; KAL-LN2E1 cells: lung squamous carcinoma cells). Hubertus Hochrein’s team applied recombinant modified vaccinia virus Ankara (rMVA)-encoded co-stimulating CD40L to treat solid tumors (mouse melanoma and lymphoma), inducing the secretion of cytokines like IL-18 and IFN-α after intravenous injection to ensure NK expansion and activation, eventually killing the tumor cells [81]. In addition, Kuang et al. found that chiral NPs strengthened the tumoricidal ability of NK cells through stimulating cytokine (IL-18 and IL-12) expression [83]. The results exhibited that the mice treated with L-type NPs induced (33.62 ± 3.41)% NK activation in tumors, which was 1.39 times higher than D-type NP groups.

### 3.3. Expression or Binding Prevention of Inhibitory Receptors’ Ligands

The stimulus-inhibitory signal transduction balance of NK cells can be effectively broken through the prevention of the NK-expressed inhibitory receptors from binding to their ligands. Meanwhile, the immunosuppressive state of NK cells can be overcome, and their anti-tumor property will be subsequently enhanced.

Blocking the binding between human leukocyte antigen-E (HLA-E) and NKG2A is beneficial to enhance NK-mediated immunotherapy against tumors. Recently, studies have shown that both inorganic and organic selenite are available to down-regulate the expression of HLA-E in tumor cells but activate NK cells. Yang et al. prepared a type of enzyme and reactive oxygen species (ROS) double-sensitive selenopeptide NP (SeP/DOX) [87]. After intravenous injection, the NPs precisely aggregated in tumors by recognizing the overexpressed αvβ3 integrin on endothelial cells in the neovascular system of MDA-MB-231 tumors. The selenopeptide in NPs was decomposed in the high-ROS TME, and the oxidative metabolite alkylselenic acid activated the immune response of NK cells by blocking the binding of HLA-E with NKG2A. Another selenium compound for NK activation was designed by Xu et al., who prepared a cytosine diselenide compound (Cyt-SeSe-Cyt) and diselenide–pemetrexed (Pem/Se) [45]. The diselenide bond in this compound broke under low-dose γ-ray irradiation to produce selenite acid, which not only inhibited the expression of HLA-E on breast cancer cells, but also blocked the NK-expressed inhibitory immune checkpoint to promote tumor eradication through NK cells. In addition, Xu and his teammates prepared PSeR/DOX NPs through the self-assembly of amphipathic polymer PSeR containing selenium and DOX [88]. In vitro experiments showed that the selenite molecules produced by radiation therapy significantly improved the anti-tumor activity of NK cells.

### 3.4. ADCC Induction of NK Cells

When the Fc receptors on tumor cells bind to the Fab fragments from monoclonal antibodies (lgG), the CD16 on NK cells could identify the Fc domains of lgG and kill the tumor, which is also known as the ADCC effect [100]. Generally, the NK-mediated ADCC effect will be largely limited by the heterogeneity of tumor antigens. This is attributed to the fact that it is difficult to identify a specific tumor antigen stably expressed on various tumors, and only specific antigens can be recognized through antibody-mediated ADCC. Thus, it is urgent to seek a suitable route to strengthen the NK-induced ADCC effect for tumor therapy.

To overcome the aforementioned limitation, Ji et al. developed a strategy that did not require the presence of specific antigens on distinct tumors (B16-F10 cells; 4T1 cells; MDA-MB-231 cells; Raji: human lymphoma cell line) to ensure tumor targetability [76]. After binding Fc fragments or therapeutic monoclonal antibodies to the N-terminal of the peptide, the pH-low insertion peptides (pHLIPs) underwent conformational transformation in the weakly acidic TME and then assembled to the membrane of tumor cells. These Fc fragments or antibodies bound to the NK-expressed CD16 to directly activate NK cells and initiate the ADCC effect for tumor eradication. Experimental results indicated that this strategy had the potential to treat various primary tumors and tumor metastases. Lu et al. designed an immunomodulatory NP (IMN) that can directly modify NK activation signals on tumor cells (B16F10 cells, 4T1 cells, and MDA-MB-231 cells) [77]. After intravenous injection, the NPs could accumulate in tumors and decompose the glucose-modified poly(2-methacryloyloxyethyl phosphorylcholine) (PMPC) shell in the TME to release the bifunctional core (nBSA-PBA-IgG). nBSA-PBA-IgG attached to the tumor cell membrane through interacting between phenylboronic acid (PBA) and sialic acid and realized the in situ modification of NK-activating ligand (IgG) on tumor cells to activate the nearby NK cells.

### 3.5. Other Strategies for NK Cell Stimulation

In addition to the above methods, studies have revealed that phosphite-modified phosphorus dendritic macromolecules can also promote NK proliferation in peripheral blood monocytes (PBMCs) by inhibiting regulatory T cells (Tregs) and regulating the signals between monocytes and NK cells [101,102]. Based on this strategy, Shi et al. constructed an immune-regulatory drug-carrying NP containing phosphorus crown macromolecules, M-G1-TBPNa@DOX [91]. The experimental results showed that the inherent immunomodulatory activity of these NP micelles promoted the proliferation of NK cells in PBMCs, which were recruited to the tumor site through blood circulation, and exerted anti-tumor efficacy in coordination with other immune cells.

In addition, researchers have also synthesized bispecific T-cell conjugates (BiTEs) and tri-specific killer conjugates (TriKEs), which effectively enhance the cytotoxicity of NK cells against tumors. These structures are typically connected by a single-chain antibody fragment (scFv) of the anti-CD16 antibody with one BiKE or two TriKEs of tumor antigen-specific antibodies’ scFv. These structures allow high-affinity CD16^+^ NK cells to bind to tumor cells, hence overcoming the limitations caused by receptor polymorphism. Felices et al. designed a TriKE of tri-specific biologic drugs containing distinct antibody fragments (including single-domain antibodies against CD16, single-chain antibodies against HER2 antigens on cancer cells, and IL-15), which could activate NK cells through different pathways [85]. These antibody fragments could simultaneously bind with both NK cells and ovarian cancer cells, and the IL-15 portion was applied to specifically mediate IL-15 signaling on NK cells when inducing ADCC effects for triggering robust NK expansion. Compared with the control group, TriKEs promoted the proliferation of NK cells and ensured the highest NK amount among all experimental groups during the entirety of tumor therapy.

**Figure 3:**
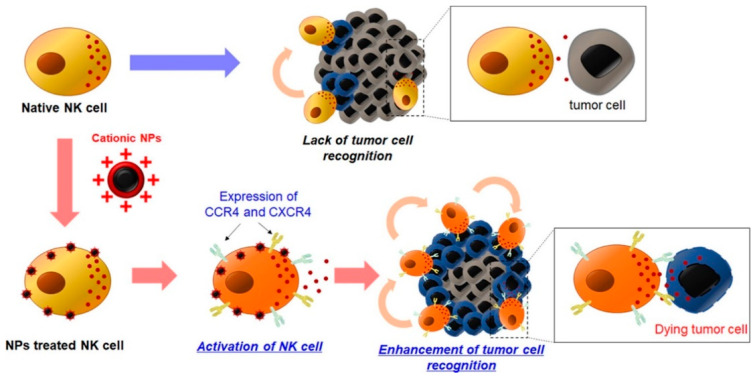
Schematic diagram of enhancing the anti-tumor effect of NK cells by cNPs. Natural NK cells cannot directly recognize tumor cells. After the stimulation of cNPs, the expression of CCR4 and CXCR4 on NK was induced and the ability of NK cells to recognize and kill tumor cells was increased. Picture reprinted from Ref. [95].

Studies have shown that NPs containing cationic polymers could promote the production of pro-inflammatory cytokines and induce a strong humoral response [103,104]. Park et al. developed a kind of cationic NP (cNP) by applying polydopamine (PDA) chemistry to immobilize PEI onto the surface of magnetic NPs for NK activation (Figure 3) [95]. cNP could increase the expression of chemokine receptors (CCR4 and CXCR4) on NK cells. In vitro experiments showed that the cytotoxicity of cNP-treated primary NK and NK-92MI cells against MDA-MB-231 cells was more than twice as high as that of untreated NK cells. Meanwhile, in vivo studies also proved that cNP-treated NK cells significantly inhibited tumor growth.

## 4. NK Cells and NK-Derived Exosomes as Drug Delivery Vehicles for Tumor Immunotherapy

NK cells possess numerous advantages in immunotherapy, including abundant cell sources [71,105], high safety profiles [106,107], reduced susceptibility to cytokine release syndrome (CRS), minimal neurotoxicity, and low immunostimulatory properties during therapy [108,109,110,111]. Moreover, NK-based immunotherapies (such as CAR-NK) have recently demonstrated satisfactory clinical outcomes [112,113,114,115]. However, the therapeutic effect of NK cells against solid tumors is usually inadequate due to insufficient local NK infiltration and NK deactivation induced by the TME [116,117]. Meanwhile, natural NK cells cannot perform targeted cancer immunotherapy due to the lack of cell-specific receptors [118]. In general, anti-tumor efficacy can be promoted with increased NK infiltration in tumors. Therefore, it is valuable to seek suitable methods to increase intratumoral NK recruitment and infiltration.

**Figure 4:**
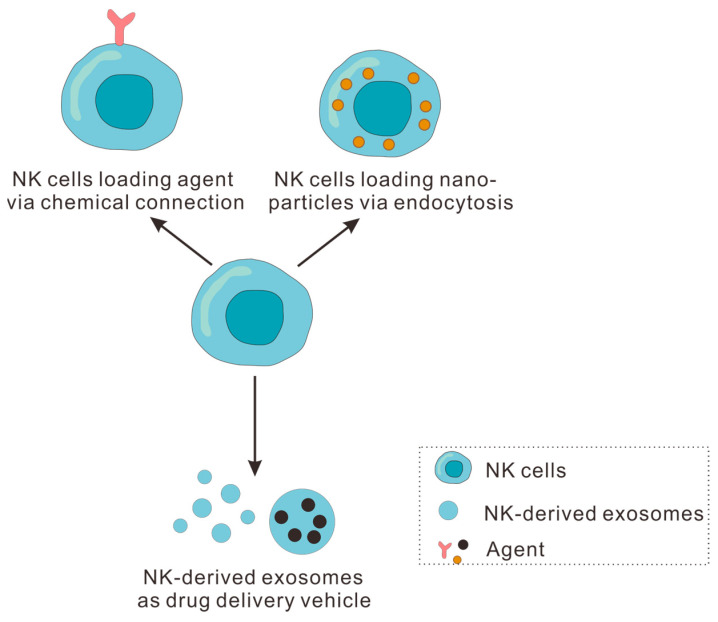
Schematic illustration of NK cells and NK-derived exosomes as drug delivery vehicles for tumor immunotherapy.

Fortunately, the emergence of DDSs provides a novel idea to solve the above problems. Recently, it has been feasible to design distinct NK-derived drug delivery platforms based on the existence of numerous modifiable groups on NK cell surfaces. According to the latest studies, NK cells have been successfully surface-modified with various antibodies and proteins [118,119]. In this section, we will focus on the application of NK cells themselves and NK-derived exosomes as drug delivery vehicles for anti-tumor immunotherapy (Figure 4).

### 4.1. NK Cells as Drug Delivery Vehicle for Tumor Immunotherapy

#### 4.1.1. NK Cell Loading Agent via Chemical Connection

Due to the poor tumor tropism of NK cells, directly administrating innate NK cells can neither reach deep tumor sites nor exhibit tumor targetability. In order to allow NK cells to be tumor-targetable, some studies have loaded NPs or other modifiers onto the cell surface through the modifiable groups on NK cells to enhance NK cells’ anti-tumor efficacy.

In past studies, a large number of NK cells have been modified with antibodies and other proteins by modifying the surface of NK cells. For example, Yang et al. modified the synthesized CD30-specific aptamer on NK surfaces to obtain engineered NK cells (ApEn-NK) [118]. ApEn-NKs were able to specifically bind to CD30-expressing lymphoma cells and had a greater capacity to kill lymphoma cells compared with NK cells by unmodified antibodies. Similarly, Li et al. modified IgG antibodies onto the glycocalyx on the NK surface to obtain Herceptin-NK-92MI, exhibiting enhanced activity in vitro, and induce the lysis and death of HER2^+^ cancer cells [119]. The basic structure of a CAR includes an antigen recognition domain, an extracellular hinge domain, a transmembrane domain, and an intracellular signal domain composed of scFv [120].

CAR-NK cells are produced using genetic engineering technology, which enables the expression of CAR on NK cells [121]. Specifically, genetic engineering technology involves integrating a chimeric antibody CAR onto NK cells, enabling them to recognize and attack tumor cells. The chimeric antibody CAR can enable CAR-NK to accurately and efficiently kill tumor cells in vivo. Compared with CAR-T cells, CAR-NK cells show a wider anti-tumor spectrum and better clinical safety (as they never induce graft versus host disease) and are less prone to cytokine release syndrome [122,123]. Recently, CAR-NK cells have been widely studied in clinical practice; for example, the China National Medical Products Administration approved the clinical trial application of IBR854 cell injection (CAR-NK injection derived from homologous peripheral blood) in 2022 for treating solid tumors. Exogenous cytokine IL-21 can not only induce continuous NK expansion and activation, but also enhances the maturation and persistence of isolated NK cells. However, low doses of IL-21 only produce weak proliferation and differentiation effects on NK cells, while overdose may cause severe toxicity effects like systemic cytokine storm, lymphocyte reduction, and an increase in transaminases. To circumvent the side effects of IL-21 during administration of the highest measure, Cai et al. implanted -N_3_/-BCN motifs on both NK and lymphoma cells by glycoconjugate engineering and coupled IL-21-containing NPs (ILNPs) on NK cells [124]. The above two cells were modified with complementary bioorthogonal chemical moieties, which could achieve effective tumor recognition and promote NK infiltration into tumor tissue. Meanwhile, the above redox NPs coupled on NK cells could continuously release cytokine IL-21 in the TME, generating an effective and sustained “pseudo-autocrine” stimulation of NK cells. In short, this strategy provided a novel idea for promoting NK proliferation and activation, enhancing the therapeutic potential, and limiting the systemic toxicity during cancer therapy. Kim et al. prepared a kind of DOX-encapsulated pH-sensitive micelle, which was linked to the surface thiol groups on NK cells via maleimide–thiol coupling chemistry to obtain RE-NK cells [125]. When the circulating NK cells approached tumor tissue together with chemokines, these NK cells initiated the formation of immune synapses (ISs), and subsequently released acidic granules to stimulate drug release. When RE-NK cells attacked tumors, the local acidic TME could realize rapid micelle disassembly and eventually ensured the sufficient release of DOX.

In addition to directly modifying NPs onto NK cells in vitro, researchers at Cornell University prepared a liposome carrying TRAIL protein, which was injected in vivo and subsequently immobilized on the NK cell surface after binding to the antibody against NK1.1 to obtain “super-natural killer cells” [126]. These “super-natural killer cells” could accumulate in tumor-draining lymph nodes for a long time, inducing the apoptosis of cancer cells and preventing the lymphatic spread of subcutaneous colon cancer in mice.

#### 4.1.2. NK Cells Loading Nanoparticles via Endocytosis

**Figure 5:**
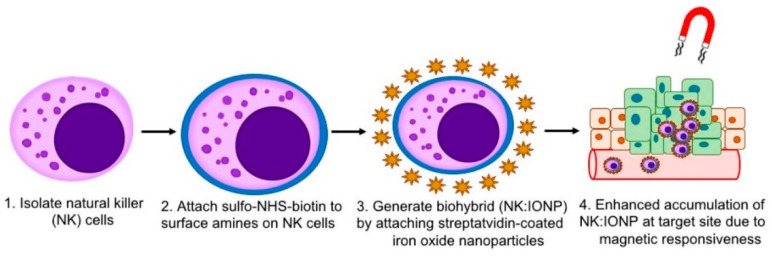
The construction and application of NK:IONP for tumor therapy, including the isolation and surface modification of NK cells, the synthesis of NK:IONP, and the tumor targeting mechanism of NK:IONP. Picture reprinted from Ref. [130].

Living NK cells possess an active NP uptake ability; thus, the loading of nanoparticles can also be achieved by active cellular uptake [127]. Based on this, Jiang et al. developed superparamagnetic iron oxide NPs (Fe_3_O_4_@PDA NPs) composed of magnetic Fe_3_O_4_ cores with PDA shells for magnetically targeted therapy [128]. Fe_3_O_4_@PDA NPs had excellent stability and biocompatibility in vivo, and they could be actively ingested by NK cells without affecting the biological function of NK cells. Both in vitro and in vivo studies demonstrated that Fe_3_O_4_@PDA NP-labeled NK cells possessed higher intratumoral aggregation with the presence of a magnetic field, which significantly inhibited the tumor growth through increasing the apoptosis of A549 cells (human non-small-cell lung cancer cells). Similarly, Cheng et al. obtained Cy5.5-conjugated Fe_3_O_4_/SiO_2_ core/shell NPs, and co-incubated them with NK-92MI cells to enter the interior of these cells via endocytosis [129]. The NP-loaded NK cells were then administrated to mice suffering from B cell lymphoma through tail vein injection, and the location of NK-92MI cells could be accurately controlled by a magnetic field. The results showed that the tumor penetration rate of NP-loaded NK-92MI was 17 times higher than that of normal NK cells under an external magnetic field, while the tumor-killing activity of NK cells was not affected. Fernandes et al. designed a magnetically responsive NK:IONP by modifying iron oxide nanoparticles (IONPs) onto NK cells (Figure 5) [130]. These engineered NK cells could be precisely delivered to the desired target tumor site under magnetic guidance, eventually achieving maximum tumor eradication efficacy.

### 4.2. NK-Derived Exosomes as Drug Delivery Vehicle for Tumor Immunotherapy

NEOs possess similar immune regulatory functions to NK cells but have better tumor permeability compared with NK cells as they have a smaller particle size (40–150 nm), making them not easily affected by the TME [131]. Clinical studies revealed that NEOs have a tumor-killing ability and can be applied as an anti-tumor agent alone [132]. NEOs also have multitudinous advantages like high safety, easy storage and transportation, and tumor targeting [133,134]. However, the anti-tumor effect of NEOs is not strong enough when they are separately adopted [135]. In order to better exert the anti-tumor ability of NEOs, their outstanding drug-loading capacity was utilized to improve their immune regulatory ability. Zhao et al. evaluated the application of extracellular vesicles derived from amplified NKs (eNK-EXO) in treating human ovarian epithelial cells (IOSE80 cells) [46]. The results indicated that eNK-EXO expressed NK-characteristic proteins and cytotoxic substances, which could be selectively ingested by ovarian cancer cells, directly inducing cell apoptosis and enhancing the cytotoxicity of NK cells with impaired function in TME. Then, the anti-tumor drug cisplatin was loaded into eNK-EXO for preparing DDP. DDP exerted a critical effect on inhibiting the proliferation of ovarian cancer cells, especially chemotherapy-resistant cells, and significantly promoted cell apoptosis.

**Figure 6:**
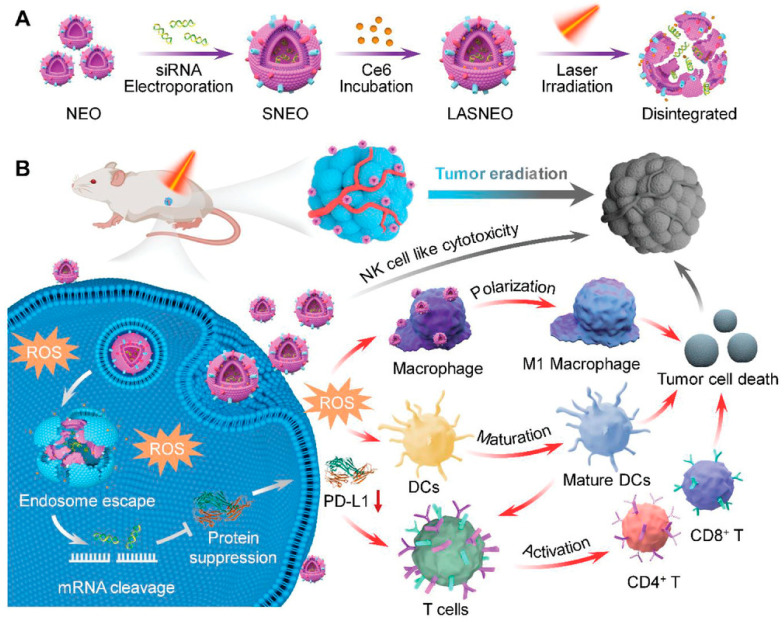
Schematic illustration of LASNEO-mediated collaborative tumor eradication. (**A**) Description of the preparation of siRNA and Ce6 dual-loaded LASNEO, and the photo-triggered LASNEO disassembly. (**B**) LASNEO collaboratively reprograms multiple types of immune cells for tumor immune stimulation. Picture reprinted from Ref. [135].

In addition to anti-tumor agents, Huang et al. loaded hydrophilic small interfering nucleic acids (siRNAs) into NEOs through electroporation, then loaded photosensitizer Ce6 onto the NEO membrane to construct light-activatable silencing NEOs (LASNEOs) (Figure 6) [135]. After entering cells, LASNEOs could produce ROS under 660 nm light irradiation, which achieved satisfactory photodynamic therapy through promoting the gene-silencing effect of siRNA after its entry and release into the cytoplasm of HepG2-Luc cells.

## 5. Summary and Outlook

In this review, we first introduced the origin, classification, and anti-tumor mechanism of NK cells, then described the activation of NK cells by DDS in tumor immunotherapy. NK cells are a type of important tumor killer cell in vivo, and the activation state of NK is determined by the balance between activation and inhibition signals on the NK surface. Tumor cells can evade the recognition of T cells due to the lack of MHC-I expression. In comparison, NK cells can directly induce the apoptosis of tumor cells without MHC restriction, which plays an important role in tumor immunity and exerts enhanced tumoricidal efficacy during treatment. NK cells are also an important source of cytokines in vivo, which can induce tumor eradication by secreting distinct cytokines (such as TNF-α, IFN-γ, IL-2, etc.). The surface of NK cells expresses a large number of signaling receptors, which can be finally classified into activating receptors (like NKG2D, KIR-2DS, NKp46, DNAM1, IL-2R, etc.) and inhibitory receptors (namely NKG2A, NKG2B, Tim-3, PD-1, etc.). With the diversity of DDSs, NK cells exert anti-tumor activity after being stimulated by regulating the balance between active and inhibitory signals of NK in vivo. NK cells can be activated through stimulating the expression of stimulatory receptors or their ligands, inhibiting the expression of NK inhibitory receptors or blocking the binding to their ligands, or loading the cytokines with NK activation properties into various DDSs to directly stimulate NK cells after administration. In addition, NK cells exhibit the advantages of easy culture and expansion in vitro, and can also be directly applied as drug delivery carriers. Modifiers or NPs are firstly loaded on NK cell surfaces to activate NK cells, and these activated NK cells can be later transfused back into the body to exert anti-tumor effects. NEOs, which possess anti-tumor efficacy and tumor targeting, are also used as drug delivery carriers, loading anti-tumor agents or RNA to synergistically exert NEOs’ anti-tumor effect.

Immunotherapy based on NK activation has indeed achieved certain promising effects in treating hematologic tumors and has brought many expectations for curing tumors. Nevertheless, its broad application against tumors in clinicals has still been limited due to the following problems: (1) Due to the insufficient infiltration of NK cells in solid tumors and the influence of the TME on NK activity, the effect of NK-based immunotherapy against solid tumors is not ideal. (2) The half-life of NK cells is relatively short, and although NK-based therapy shows higher safety compared with T cells, NK-based immunotherapy requires more repeated administration to ensure treatment efficacy. (3) Due to the lack of cell-specific receptors, NK cells cannot directly target cancer sites. Although there have been some studies on modifying NK cells to make them more effective in tumor targeting, modified NK cells have not been involved in clinical studies in treating solid tumors. In brief, because of the above shortcomings of NK-based therapies, it remains necessary to seek more suitable solutions before further clinical application to cancer therapy.

Despite the fact that there are various problems that need urgently addressing in NK-based tumor therapy, with the progress of biomedical technology and the further research on anti-tumor treatment, we believe that these current obstacles in the development of immunotherapy based on NK activation can be overcome in the future. Meanwhile, the essentiality of NK therapy in tumor treatment will also be ultimately improved.

## Figures and Tables

**Table 1:** Examples of various strategies for NK activation.

Target Receptors on NK Cells	Nanoparticles	Mechanisms of NK Activation	Ref.
CD16	pHLIP-Fc or pHLIP-mAb	Inducing ADCC effect	[76]
	IMN	Inducing ADCC effect	[77]
IL-2R	NR_P+I_	Delivering IL-2 (immunotherapeutic agent)	[78]
IL-12R	RDMCM/pIL-12 + PD-1/PD-L1i	Delivering IL-12 encoding plasmid	[79]
IL-15R	IL-15-NPs	Delivering IL-15 (immunotherapeutic agent)	[80]
IL-18R	MVA-CD40L	Activating the secretion of IL-18, IFN-α, etc.	[81]
IL-6R and IL-12R	PRINT-CpG	Activating the secretion of IFN-γ, IL-6, IL-12, etc.	[82]
IL-12R and IL-18R	D- or L-type NPs	Activating the secretion of IL-18 and IL-12	[83]
IL-12R and IL-27R	DAL4-LNP-IL-12 + IL-27 mRNA	Delivering IL-12 and IL-27 mRNA	[84]
IL-15R and CD16	TriKEs	Delivering IL-15 and inducing ADCC effect	[85]
Multiple Cytokine receptor	hMSC-DPP	Activating the secretion of IFN-γ, IL-2, IL-4, IL-12, GM-CSF, etc.	[86]
NKG2A	SeP/DOX	Blocking the binding between HLA-E and NKG2A	[87]
	Pem/Se	Blocking the binding between HLA-E and NKG2A	[45]
	PSeR/DOX NPs	Blocking the binding between HLA-E and NKG2A	[88]
NKG2D	ZCMF	Upregulating the expression of NKG2D ligands	[43]
	RuPOP	Upregulating the expression of NKG2D ligands	[89]
	CD@MSN	Upregulating the expression of NKG2D ligands	[90]
Undefined	M-G1-TBPNa@DOX	Inhibiting the regulatory T cells and regulating the signaling between NK and monocytes	[91]
	PF_11_DG	Activating the cytotoxicity of NK cells	[92]
	MNGs	Activating the cytotoxicity of NK cells	[93]
	Zn-LDH	Neutralizing tumor acidity to restore the function of NK cells	[94]
	cNPs	Increasing the expression of CCR4 and CXCR4 on NK	[95]

## Abbreviations

ADCC	Antibody-dependent cell-mediated cytotoxicity
APCs	Antigen-presenting cells
CRS	Cytokine release syndrome
DDS	Drug delivery system
HLA-E	Human leukocyte antigen-E
IFN-γ	Interferon-γ
IL-12R	Interleukin 12 receptor
IL-15	Interleukin 15
IL-15R	Interleukin 15 receptor
IL-18R	Interleukin 18 receptor
IL-21R	Interleukin 21 receptor
IL-2R	Interleukin 2 receptor
ISs	Immune synapses
KIRs	Killer cell immunoglobulin-like receptors
MHC	Major histocompatibility complex
MHT	Mild magnetothermal therapy
NCAM	Neural cell adhesion molecule
NCR1	Natural cytotoxic triggering receptor 1
NEO	NK-derived exosomes
NK	Natural killer
NKG2A	Natural Killer Group 2A
PBMCs	Peripheral blood monocytes
PD-1	Programmed death 1
PDA	Polydopamine
PS	Phosphatidylserine
ROS	Reactive oxygen species
siRNAs	Small interfering nucleic acids
Tim-3	T-cell immunoglobulin and mucin domain Containing protein-3
TNFR	Tumor necrosis factor receptor
TNF-α	Tumor necrosis factor-α
Tregs	Inhibiting regulatory T cells
ULBPs	UL16-binding proteins

## References

[1] Siegel R.L., Miller K.D., Wagle N.S., Jemal A. (2023). Cancer statistics, 2023. CA-Cancer J. Clin..

[2] Tohme S., Simmons R.L., Tsung A. (2017). Surgery for cancer: A trigger for metastases. Cancer Res..

[3] Pardoll D.M. (2012). The blockade of immune checkpoints in cancer immunotherapy. Nat. Rev. Cancer.

[4] Barker H.E., Paget J.T.E., Khan A.A., Harrington K.J. (2015). The tumour microenvironment after radiotherapy: Mechanisms of resistance and recurrence. Nat. Rev. Cancer.

[5] DeVita V.T., Chu E. (2008). A history of cancer chemotherapy. Cancer Res..

[6] Sullivan R., Alatise O.I., Anderson B.O., Audisio R., Autier P., Aggarwal A., Balch C., Brennan M.F., Dare A., D’Cruz A. (2015). Global cancer surgery: Delivering safe, aff ordable, and timely cancer surgery. Lancet Oncol..

[7] Strobel O., Neoptolemos J., Jaeger D., Buechler M.W. (2019). Optimizing the outcomes of pancreatic cancer surgery. Nat. Rev. Clin. Oncol..

[8] Bukowski K., Kciuk M., Kontek R. (2020). Mechanisms of multidrug resistance in cancer chemotherapy. Int. J. Mol. Sci..

[9] Schaue D., McBride W.H. (2015). Opportunities and challenges of radiotherapy for treating cancer. Nat. Rev. Clin. Oncol..

[10] Li B., Shao H., Gao L., Li H., Sheng H., Zhu L. (2022). Nano-drug co-delivery system of natural active ingredients and chemotherapy drugs for cancer treatment: A review. Drug Deliv..

[11] Liu Y.-Q., Wang X.-L., He D.-H., Cheng Y.-X. (2021). Protection against chemotherapy- and radiotherapy-induced side effects: A review based on the mechanisms and therapeutic opportunities of phytochemicals. Phytomedicine.

[12] Goldberg M.S. (2019). Improving cancer immunotherapy through nanotechnology. Nat. Rev. Cancer.

[13] Gowd V., Ahmad A., Tarique M., Suhail M., Zughaibi T.A., Tabrez S., Khan R. (2022). Advancement of cancer immunotherapy using nanoparticles-based nanomedicine. Semin. Cancer Biol..

[14] Mei Y., Tang L., Zhang L., Hu J., Zhang Z., He S., Zang J., Wang W. (2022). A minimally designed PD-L1-targeted nanocomposite for positive feedback-based multimodal cancer therapy. Mater. Today.

[15] Peng S., Xiao F., Chen M., Gao H. (2022). Tumor-microenvironment-responsive nanomedicine for enhanced cancer immunotherapy. Adv. Sci..

[16] Sharma P., Hu-Lieskovan S., Wargo J.A., Ribas A. (2017). Primary, adaptive, and acquired resistance to cancer immunotherapy. Cell.

[17] Sivori S., Pende D., Quatrini L., Pietra G., Della Chiesa M., Vacca P., Tumino N., Moretta F., Mingari M.C., Locatelli F. (2021). NK cells and ILCs in tumor immunotherapy. Mol. Asp. Med..

[18] Seidel U.J.E., Schlegel P., Lang P. (2013). Natural killer cell mediated antibody-dependent cellular cytotoxicity in tumor immunotherapy with therapeutic antibodies. Front. Immunol..

[19] Kiessling R., Klein E., Wigzell H. (1975). “Natural” killer cells in the mouse. I. cytotoxic cells with specificity for mouse moloney leukemia cells. specificity and distribution according to genotype. Eur. J. Immunol..

[20] Herberman R.B., Nunn M.E., Lavrin D.H. (1975). Natural cytotoxic reactivity of mouse lymphoid cells against syngeneic acid allogeneic tumors. I. Distribution of reactivity and specificity. Int. J. Cancer.

[21] Liu S., Galat V., Galat Y., Lee Y.K.A., Wainwright D., Wu J. (2021). NK cell-based cancer immunotherapy: From basic biology to clinical development. J. Hematol. Oncol..

[22] Mestre-Duran C., Martin-Cortazar C., Garcia-Solis B., Pernas A., Pertinez L., Galan V., Sisinni L., Clares-Villa L., Navarro-Zapata A., Al-Akioui K. (2023). Ruxolitinib does not completely abrogate the functional capabilities of TLR4/9 ligand-activated NK cells. Front. Immunol..

[23] Lanier L.L., Testi R., Bindl J., Phillips J.H. (1989). Identity of Leu-19 (CD56) leukocyte differentiation antigen and neural cell adhesion molecule. J. Exp. Med..

[24] Shimasaki N., Jain A., Campana D. (2020). NK cells for cancer immunotherapy. Nat. Rev. Drug Discov..

[25] Freud A.G., Zhao S., Wei S., Gitana G.M., Molina-Kirsch H.F., Atwater S.K., Natkunam Y. (2013). Expression of the activating receptor, NKp46 (CD335), in human natural killer and T-cell neoplasia. Am. J. Clin. Pathol..

[26] Pende D., Falco M., Vitale M., Cantoni C., Vitale C., Munari E., Bertaina A., Moretta F., Del Zotto G., Pietra G. (2019). Killer Ig-like receptors (KIRs): Their role in NK cell modulation and developments leading to their clinical exploitation. Front. Immunol..

[27] Cong J., Wei H. (2019). Natural killer cells in the lungs. Front. Immunol..

[28] Wagner J.A., Rosario M., Romee R., Berrien-Elliott M.M., Schneider S.E., Leong J.W., Sullivan R.P., Jewell B.A., Becker-Hapak M., Schappe T. (2017). CD56^bright^ NK cells exhibit potent antitumor responses following IL-15 priming. J. Clin. Investig..

[29] Prager I., Liesche C., van Ooijen H., Urlaub D., Verron Q., Sandstrom N., Fasbender F., Claus M., Eils R., Beaudouin J. (2019). NK cells switch from granzyme B to death receptor-mediated cytotoxicity during serial killing. J. Exp. Med..

[30] Yu Y. (2023). The function of NK cells in tumor metastasis and NK cell-based immunotherapy. Cancers.

[31] Morvan M.G., Lanier L.L. (2016). NK cells and cancer: You can teach innate cells new tricks. Nat. Rev. Cancer.

[32] Zamai L., Ponti C., Mirandola P., Gobbi G., Papa S., Galeotti L., Cocco L., Vitale M. (2007). NK cells and cancer. J. Immunol..

[33] Lyu M.A., Rosenblum M.G. (2005). The immunocytokine scFv23/TNF sensitizes HER-2/neu-overexpressing SKBR-3 cells to tumor necrosis factor (TNF) via up-regulation of TNF receptor-1. Mol. Cancer Ther..

[34] Ebert L.M., Meuter S., Moser B. (2006). Homing and function of human skin γδ T cells and NK cells:: Relevance for tumor surveillance. J. Immunol..

[35] Owen C.A., Campbell M.A., Boukedes S.S., Campbell E.J. (1995). Inducible binding of bioactive cathepsin-G to the cell-surface of neutrophils—A novel mechanism for mediating extracellular catalytic activity of cathepsin-G. J. Immunol..

[36] Aqbi H.F., Wallace M., Sappal S., Payne K.K., Manjili M.H. (2018). IFN-γ orchestrates tumor elimination, tumor dormancy, tumor escape, and progression. J. Leukoc. Biol..

[37] Klingemann H., Boissel L., Toneguzzo F. (2016). Natural killer cells for immunotherapy-advantages of the NK-92 cell line over blood NK cells. Front. Immunol..

[38] Wang W., Erbe A.K., Hank J.A., Morris Z.S., Sondel P.M. (2015). NK cell-mediated antibody-dependent cellular cytotoxicity in cancer immunotherapy. Front. Immunol..

[39] He Y., Xiong T., He S., Sun H., Huang C., Ren X., Wu L., Patterson L.H., Zhang J. (2021). Pulmonary targeting crosslinked cyclodextrin metal-organic frameworks for lung cancer therapy. Adv. Funct. Mater..

[40] Zhu T., Chen Z., Jiang G., Huang X. (2023). Sequential targeting hybrid nanovesicles composed of chimeric antigen receptor T-cell-derived exosomes and liposomes for enhanced cancer immunochemotherapy. Acs Nano.

[41] Liau L.M., Ashkan K., Tran D.D., Campian J.L., Trusheim J.E., Cobbs C.S., Heth J.A., Salacz M., Taylor S., D’Andre S.D. (2018). First results on survival from a large Phase 3 clinical trial of an autologous dendritic cell vaccine in newly diagnosed glioblastoma. J. Transl. Med..

[42] Huang D., Wang Y., Yang F., Shen H., Weng Z., Wu D. (2017). Charge-reversible and pH-responsive biodegradable micelles and vesicles from linear-dendritic supramolecular amphiphiles for anticancer drug delivery. Polym. Chem..

[43] Pan J., Xu Y., Wu Q., Hu P., Shi J. (2021). Mild magnetic hyperthermia-activated innate immunity for liver cancer therapy. J. Am. Chem. Soc..

[44] Gauthier L., Morel A., Anceriz N., Rossi B., Blanchard-Alvarez A., Grondin G., Trichard S., Cesari C., Sapet M., Bosco F. (2019). Multifunctional natural killer cell engagers targeting nkp46 trigger protective tumor immunity. Cell.

[45] Li T., Pan S., Gao S., Xiang W., Sun C., Cao W., Xu H. (2020). Diselenide-pemetrexed assemblies for combined cancer immuno-, radio-, and chemotherapies. Angew. Chem. Int. Ed..

[46] Luo H., Zhou Y., Zhang J., Zhang Y., Long S., Lin X., Yang A., Duan J., Yang N., Yang Z. (2023). NK cell-derived exosomes enhance the anti-tumor effects against ovarian cancer by delivering cisplatin and reactivating NK cell functions. Front. Immunol..

[47] Zmievskaya E.A., Mukhametshin S.A., Ganeeva I.A., Gilyazova E.M., Siraeva E.T., Kutyreva M.P., Khannanov A.A., Yuan Y., Bulatov E.R. (2024). Artificial Extracellular Vesicles Generated from T Cells Using Different Induction Techniques. Biomedicines.

[48] Klein L., Kyewski B., Allen P.M., Hogquist K.A. (2014). Positive and negative selection of the T cell repertoire: What thymocytes see (and don’t see). Nat. Rev. Immunol..

[49] Roche P.A., Furuta K. (2015). The ins and outs of MHC class II-mediated antigen processing and presentation. Nat. Rev. Immunol..

[50] Merino A., Maakaron J., Bachanova V. (2023). Advances in NK cell therapy for hematologic malignancies: NK source, persistence and tumor targeting. Blood Rev..

[51] Han B., Song Y., Park J., Doh J. (2022). Nanomaterials to improve cancer immunotherapy based on ex vivo engineered T cells and NK cells. J. Control. Release.

[52] Carotta S. (2016). Targeting NK cells for anticancer immunotherapy: Clinical and preclinical approaches. Front. Immunol..

[53] Yang L., Yang Y., Chen Y., Xu Y., Peng J. (2022). Cell-based drug delivery systems and their in vivo fate. Adv. Drug Deliver. Rev..

[54] Vivier E., Tomasello E., Paul P. (2002). Lymphocyte activation via NKG2D: Towards a new paradigm in immune recognition?. Curr. Opin. Immunol..

[55] Gong Y., Klein Wolterink R.G.J., Wang J., Bos G.M.J., Germeraad W.T.V. (2021). Chimeric antigen receptor natural killer (CAR-NK) cell design and engineering for cancer therapy. J. Hematol. Oncol..

[56] Koene H.R., Kleijer M., Algra J., Roos D., vondemBorne A., deHaas M. (1997). Fc gamma RIIIa-158V/F polymorphism influences the binding of IgG by natural killer cell Fc gamma RIIIa, independently of the Fc gamma IIIa-48L/R/H phenotype. Blood.

[57] Brandt C.S., Baratin M., Yi E.C., Kennedy J., Gao Z., Fox B., Haldeman B., Ostrander C.D., Kaifu T., Chabannon C. (2009). The B7 family member B7-H6 is a tumor cell ligand for the activating natural killer cell receptor NKp30 in humans. J. Exp. Med..

[58] Barrow A.D., Martin C.J., Colonna M. (2019). The natural cytotoxicity receptors in health and disease. Front. Immunol..

[59] Satoh-Takayama N., Dumoutier L., Lesjean-Pottier S., Ribeiro V.S.G., Mandelboim O., Renauld J.-C., Vosshenrich C.A.J., Di Santo J.P. (2009). The natural cytotoxicity receptor NKp46 is dispensable for IL-22-mediated innate intestinal immune defense against citrobacter rodentium. J. Immunol..

[60] Fuchs A., Cella M., Kondo T., Colonna M. (2005). Paradoxic inhibition of human natural interferon-producing cells by the activating receptor NKp44. Blood.

[61] Cho D., Campana D. (2009). Expansion and activation of natural killer cells for cancer immunotherapy. Korean J. Lab. Med..

[62] Cheng M., Chen Y., Xiao W., Sun R., Tian Z. (2013). NK cell-based immunotherapy for malignant diseases. Cell. Mol. Immunol..

[63] Tomala J., Chmelova H., Mrkvan T., Rihova B., Kovar M. (2009). In vivo expansion of activated naive CD8(+) T cells and NK cells driven by complexes of IL-2 and anti-IL-2 monoclonal antibody as novel approach of cancer immunotherapy. J. Immunol..

[64] Hydes T., Noll A., Salinas-Riester G., Abuhilal M., Armstrong T., Hamady Z., Primrose J., Takhar A., Walter L., Khakoo S.I. (2018). IL-12 and IL-15 induce the expression of CXCR6 and CD49a on peripheral natural killer cells. Immun. Inflamm. Dis..

[65] Nguyen K.B., Salazar-Mather T.P., Dalod M.Y., Van Deusen J.B., Wei X.Q., Liew F.Y., Caligiuri M.A., Durbin J.E., Biron C.A. (2002). Coordinated and distinct roles for IFN-alpha beta, IL-12, and IL-15 regulation of NK cell responses to viral infection. J. Immunol..

[66] Mamessier E., Sylvain A., Thibult M.-L., Houvenaeghel G., Jacquemier J., Castellano R., Goncalves A., Andre P., Romagne F., Thibault G. (2011). Human breast cancer cells enhance self tolerance by promoting evasion from NK cell antitumor immunity. J. Clin. Investig..

[67] Hiam-Galvez K.J., Allen B.M., Spitzer M.H. (2021). Systemic immunity in cancer. Nat. Rev. Cancer.

[68] Zhang Z., Wu N., Lu Y., Davidson D., Colonna M., Veillette A. (2015). DNAM-1 controls NK cell activation via an ITT-like motif. J. Exp. Med..

[69] Long E.O. (2008). Negative signaling by inhibitory receptors: The NK cell paradigm. Immunol. Rev..

[70] Cozar B., Greppi M., Carpentier S., Narni-Mancinelli E., Chiossone L., Vivier E. (2021). Tumor-infiltrating natural killer cells. Cancer Discov..

[71] Myers J.A., Miller J.S. (2021). Exploring the NK cell platform for cancer immunotherapy. Nat. Rev. Clin. Oncol..

[72] Angelo L.S., Banerjee P.P., Monaco-Shawver L., Rosen J.B., Makedonas G., Forbes L.R., Mace E.M., Orange J.S. (2015). Practical NK cell phenotyping and variability in healthy adults. Immunol. Res..

[73] Gray M.A., Stanczak M.A., Mantuano N.R., Xiao H., Pijnenborg J.F.A., Malaker S.A., Miller C.L., Weidenbacher P.A., Tanzo J.T., Ahn G. (2020). Targeted glycan degradation potentiates the anticancer immune response in vivo. Nat. Chem. Biol..

[74] Pesce S., Greppi M., Tabellini G., Rampinelli F., Parolini S., Olive D., Moretta L., Moretta A., Marcenaro E. (2017). Identification of a subset of human natural killer cells expressing high levels of programmed death 1: A phenotypic and functional characterization. J. Allergy Clin. Immun..

[75] Yang X., Li M., Qin X., Tan S., Du L., Ma C., Li M. (2022). Photophosphatidylserine guides natural killer cell photoimmunotherapy via Tim-3. J. Am. Chem. Soc..

[76] Ji T., Lang J., Ning B., Qi F., Wang H., Zhang Y., Zhao R., Yang X., Zhang L., Li W. (2019). Enhanced natural killer cell immunotherapy by rationally assembling Fc fragments of antibodies onto tumor membranes. Adv. Mater..

[77] Zheng C., Wang Q., Wang Y., Zhao X., Gao K., Liu Q., Zhao Y., Zhang Z., Zheng Y., Cao J. (2019). In situ modification of the tumor cell surface with immunomodulating nanoparticles for effective suppression of tumor growth in mice. Adv. Mater..

[78] Song Q., Yin Y., Shang L., Wu T., Zhang D., Kong M., Zhao Y., He Y., Tan S., Guo Y. (2017). Tumor microenvironment responsive nanogel for the combinatorial antitumor effect of chemotherapy and immunotherapy. Nano Lett..

[79] Hu Y., Liu X., Ran M., Yang T., Li T., Wu Y., Lin Y., Qian Z., Gao X. (2022). Simultaneous delivery of immune stimulatory gene and checkpoint blocker via targeted nanoparticles to strengthen antitumor immunity. Mater. Today Nano.

[80] Jiang D., Gao T., Liang S., Mu W., Fu S., Liu Y., Yang R., Zhang Z., Liu Y., Zhang N. (2021). Lymph lymph node delivery strategy enables the activation of cytotoxic T lymphocytes and natural killer cells to augment cancer immunotherapy. Acs Appl. Mater. Inter..

[81] Medina-Echeverz J., Hinterberger M., Testori M., Geiger M., Giessel R., Bathke B., Kassub R., Graebnitz F., Fiore G., Wennier S.T. (2019). Synergistic cancer immunotherapy combines MVA-CD40L induced innate and adaptive immunity with tumor targeting antibodies. Nat. Commun..

[82] Perry J.L., Tian S., Sengottuvel N., Harrison E.B., Gorentla B.K., Kapadia C.H., Cheng N., Luft J.C., Ting J.P.-Y., DeSimone J.M. (2020). Pulmonary delivery of nanoparticle-bound toll-like receptor 9 agonist for the treatment of metastatic lung cancer. Acs Nano.

[83] Wang W., Zhao J., Hao C., Hu S., Chen C., Cao Y., Xu Z., Guo J., Xu L., Sun M. (2022). The development of chiral nanoparticles to target NK cells and CD8(+) T cells for cancer immunotherapy. Adv. Mater..

[84] Liu J.-Q., Zhang C., Zhang X., Yan J., Zeng C., Talebian F., Lynch K., Zhao W., Hou X., Du S. (2022). Intratumoral delivery of IL-12 and IL-27 mRNA using lipid nanoparticles for cancer immunotherapy. J. Control. Release.

[85] Vallera D.A., Oh F., Kodal B., Hinderlie P., Geller M.A., Miller J.S., Felices M. (2021). A HER2 Tri-specific NK cell engager mediates efficient targeting of human ovarian cancer. Cancers.

[86] Kim K.S., Lee J.Y., Han J., Hwang H.S., Lee J., Na K. (2019). Local immune-triggered surface-modified stem cells for solid tumor immunotherapy. Adv. Funct. Mater..

[87] Wei Z., Yi Y., Luo Z., Gong X., Jiang Y., Hou D., Zhang L., Liu Z., Wang M., Wang J. (2022). Selenopeptide nanomedicine activates natural killer cells for enhanced tumor chemoimmunotherapy. Adv. Mater..

[88] Gao S., Li T., Guo Y., Sun C., Xianyu B., Xu H. (2020). Selenium-containing nanoparticles combine the NK cells mediated immunotherapy with radiotherapy and chemotherapy. Adv. Mater..

[89] Chen Q., He L., Li X., Xu L., Chen T. (2022). Ruthenium complexes boost NK cell immunotherapy via sensitizing triple-negative breast cancer and shaping immuno-microenvironment. Biomaterials.

[90] Qian M., Chen L., Du Y., Jiang H., Huo T., Yang Y., Guo W., Wang Y., Huang R. (2019). Biodegradable mesoporous silica achieved via carbon nanodots-incorporated framework swelling for debris-mediated photothermal synergistic immunotherapy. Nano Lett..

[91] Zhan M., Qiu J., Fan Y., Chen L., Guo Y., Wang Z., Li J., Majoral J.-P., Shi X. (2023). Phosphorous dendron micelles as a nanomedicine platform for cooperative tumor chemoimmunotherapy via synergistic modulation of immune cells. Adv. Mater..

[92] Wang Z., Gong X., Li J., Wang H., Xu X., Li Y., Sha X., Zhang Z. (2021). Oxygen-delivering polyfluorocarbon nanovehicles improve tumor oxygenation and potentiate photodynamic-mediated antitumor immunity. Acs Nano.

[93] Li J., Wu Y., Wang J., Xu X., Zhang A., Li Y., Zhang Z. (2023). Macrophage membrane-coated nano- gemcitabine promotes lymphocyte infiltration and synergizes AntiPD-L1 to restore the tumoricidal function. Acs Nano.

[94] Zhang L., Zhao J., Hu X., Wang C., Jia Y., Zhu C., Xie S., Lee J., Li F., Ling D. (2022). A peritumorally injected immunomodulating adjuvant elicits robust and safe metalloimmunotherapy against solid tumors. Adv. Mater..

[95] Kim K.-S., Han J.-H., Choi S.H., Jung H.-Y., Park J.D., An H.-J., Kim S.-E., Kim D.-H., Doh J., Han D.K. (2020). Cationic nanoparticle-mediated activation of natural killer cells for effective cancer immunotherapy. Acs Appl. Mater. Inter..

[96] Hellstrand K., Hermodsson S., Naredi P., Mellqvist U.H., Brune M. (1998). Histamine and cytokine therapy. Acta Oncol..

[97] Liao N., Su L., Zheng Y., Zhao B., Wu M., Zhang D., Yang H., Liu X., Song J. (2021). In vivo tracking of cell viability for adoptive natural killer cell-based immunotherapy by ratiometric NIR-II fluorescence imaging. Angew. Chem. Int. Edit..

[98] Khushalani N.I., Diab A., Ascierto P.A., Larkin J., Sandhu S., Sznol M., Koon H.B., Jarkowski A., Zhou M., Statkevich P. (2020). Bempegaldesleukin plus nivolumab in untreated, unresectable or metastatic melanoma: Phase III PIVOT IO 001 study design. Future Oncol..

[99] Dubois S.P., Miljkovic M.D., Fleisher T.A., Pittaluga S., Hsu-Albert J., Bryant B.R., Petrus M.N., Perera L.P., Muller J.R., Shih J.H. (2021). Short-course IL-15 given as a continuous infusion led to a massive expansion of effective NK cells: Implications for combination therapy with antitumor antibodies. J. Immunother. Cancer.

[100] Lin C.-W., Tsai M.-H., Li S.-T., Tsai T.-I., Chu K.-C., Liu Y.-C., Lai M.-Y., Wu C.-Y., Tseng Y.-C., Shivatare S.S. (2015). A common glycan structure on immunoglobulin G for enhancement of effector functions. Proc. Natl. Acad. Sci. USA.

[101] Poupot M., Turrin C.-O., Caminade A.-M., Fournie J.-J., Attal M., Poupot R., Fruchon S. (2016). Poly(phosphorhydrazone) dendrimers: Yin and yang of monocyte activation for human NK cell amplification applied to immunotherapy against multiple myeloma. Nanomed.-Nano Technol..

[102] Portevin D., Poupot M., Rolland O., Turrin C.-O., Fournie J.-J., Majoral J.-P., Caminade A.-M., Poupot R. (2009). Regulatory activity of azabisphosphonate-capped dendrimers on human CD4(+) T cell proliferation enhances ex-vivo expansion of NK cells from PBMCs for immunotherapy. J. Transl. Med..

[103] Yim H., Park W., Kim D., Fahmy T.M., Na K. (2014). A self-assembled polymeric micellar immunomodulator for cancer treatment based on cationic amphiphilic polymers. Biomaterials.

[104] Mulens-Arias V., Rojas J.M., Perez-Yaguee S., Morales M.P., Barber D.F. (2015). Polyethylenimine-coated SPIONs trigger macrophage activation through TLR-4 signaling and ROS production and modulate podosome dynamics. Biomaterials.

[105] Grote S., Urena-Bailen G., Chan K.C.-H., Baden C., Mezger M., Handgretinger R., Schleicher S. (2021). In vitro evaluation of CD276-CAR NK-92 functionality, migration and invasion potential in the presence of immune inhibitory factors of the tumor microenvironment. Cells.

[106] Surace L., Doisne J.-M., Escoll P., Marie S., Dardalhon V., Croft C., Thaller A., Topazio D., Sparaneo A., Cama A. (2021). Polarized mitochondria as guardians of NK cell fitness. Blood Adv..

[107] Mitwasi N., Feldmann A., Arndt C., Koristka S., Berndt N., Jureczek J., Loureiro L.R., Bergmann R., Mathe D., Hegedues N. (2020). “UniCAR”-modified off-the-shelf NK-92 cells for targeting of GD2-expressing tumour cells. Sci. Rep..

[108] Wang Z., Han W. (2018). Biomarkers of cytokine release syndrome and neurotoxicity related to CAR-T cell therapy. Biomark. Res..

[109] Porter D., Frey N., Wood P.A., Weng Y., Grupp S.A. (2018). Grading of cytokine release syndrome associated with the CAR T cell therapy tisagenlecleucel. J. Hematol. Oncol..

[110] Martinet L., Smyth M.J. (2015). Balancing natural killer cell activation through paired receptors. Nat. Rev. Immunol..

[111] Xie G., Dong H., Liang Y., Ham J.D., Rizwan R., Chen J. (2020). CAR-NK cells: A promising cellular immunotherapy for cancer. Ebiomedicine.

[112] Liu E., Marin D., Banerjee P., Macapinlac H.A., Thompson P., Basar R., Kerbauy L.N., Overman B., Thall P., Kaplan M. (2020). Use of CAR-transduced natural killer cells in CD19-positive lymphoid tumors. N. Engl. J. Med..

[113] Papayannakos C., Daniel R. (2013). Understanding lentiviral vector chromatin targeting: Working to reduce insertional mutagenic potential for gene therapy. Gene Ther..

[114] Chang Y.-H., Connolly J., Shimasaki N., Mimura K., Kono K., Campana D. (2013). A chimeric receptor with NKG2D specificity enhances natural killer cell activation and killing of tumor cells. Cancer Res..

[115] Shimasaki N., Fujisaki H., Cho D., Masselli M., Lockey T., Eldridge P., Leung W., Campana D. (2012). A clinically adaptable method to enhance the cytotoxicity of natural killer cells against B-cell malignancies. Cytotherapy.

[116] Tong L., Jimenez-Cortegana C., Tay A.H.M., Wickstrom S., Galluzzi L., Lundqvist A. (2022). NK cells and solid tumors: Therapeutic potential and persisting obstacles. Molecular Cancer.

[117] Teng K.-Y., Mansour A.G., Zhu Z., Li Z., Tian L., Ma S., Xu B., Lu T., Chen H., Hou D. (2022). Off-the-shelf prostate stem cell antigen-directed chimeric antigen receptor natural killer cell therapy to treat pancreatic cancer. Gastroenterology.

[118] Yang S., Wen J., Li H., Xu L., Liu Y., Zhao N., Zeng Z., Qi J., Jiang W., Han W. (2019). Aptamer-engineered natural killer cells for cell-specific adaptive immunotherapy. Small.

[119] Li J., Chen M., Liu Z., Zhang L., Felding B.H., Moremen K.W., Lauvau G., Abadier M., Ley K., Wu P. (2018). A single-step chemoenzymatic reaction for the construction of antibody-cell conjugates. Acs Cent. Sci..

[120] Yilmaz A., Cui H., Caligiuri M.A., Yu J. (2020). Chimeric antigen receptor-engineered natural killer cells for cancer immunotherapy. J. Hematol. Oncol..

[121] Simonetta F., Alvarez M., Negrin R.S. (2017). Natural killer cells in graft-versus-host-disease after allogeneic hematopoietic cell transplantation. Front. Immunol..

[122] Zhang L., Meng Y., Feng X., Han Z. (2022). CAR-NK cells for cancer immunotherapy: From bench to bedside. Biomark. Res..

[123] Quintarelli C., Sivori S., Caruso S., Carlomagno S., Falco M., Boffa I., Orlando D., Guercio M., Abbaszadeh Z., Sinibaldi M. (2020). Efficacy of third-party chimeric antigen receptor modified peripheral blood natural killer cells for adoptive cell therapy of B-cell precursor acute lymphoblastic leukemia. Leukemia.

[124] Meng D., Pan H., He W., Jiang X., Liang Z., Zhang X., Xu X., Wang Z., Zheng J., Gong P. (2022). In situ activated NK cell as bio-orthogonal targeted live-cell nanocarrier augmented solid tumor immunotherapy. Adv. Funct. Mater..

[125] Im S., Jang D., Saravanakumar G., Lee J., Kang Y., Lee Y.M., Lee J., Doh J., Yang Z.Y., Jang M.H. (2020). Harnessing the formation of natural killer-tumor cell immunological synapses for enhanced therapeutic effect in solid tumors. Adv. Mater..

[126] Chandrasekaran S., Chan M.F., Li J., King M.R. (2016). Super natural killer cells that target metastases in the tumor draining lymph nodes. Biomaterials.

[127] Xue J., Zhao Z., Zhang L., Xue L., Shen S., Wen Y., Wei Z., Wang L., Kong L., Sun H. (2017). Neutrophil-mediated anticancer drug delivery for suppression of postoperative malignant glioma recurrence. Nat. Nanotechnol..

[128] Wu L., Zhang F., Wei Z., Li X., Zhao H., Lv H., Ge R., Ma H., Zhang H., Yang B. (2018). Magnetic delivery of Fe_3_O_4_@polydopamine nanoparticle-loaded natural killer cells suggest a promising anticancer treatment. Biomater. Sci..

[129] Jang E.-S., Shin J.-H., Ren G., Park M.-J., Cheng K., Chen X., Wu J.C., Sunwoo J.B., Cheng Z. (2012). The manipulation of natural killer cells to target tumor sites using magnetic nanoparticles. Biomaterials.

[130] Burga R.A., Khan D.H., Agrawal N., Bollard C.M., Fernandes R. (2019). Designing magnetically responsive biohybrids composed of cord blood-derived natural killer cells and iron oxide nanoparticles. Bioconjugate Chem..

[131] Greening D.W., Gopal S.K., Xu R., Simpson R.J., Chen W. (2015). Exosomes and their roles in immune regulation and cancer. Semin. Cell Dev. Biol..

[132] Wang G., Hu W., Chen H., Shou X., Ye T., Xu Y. (2019). Cocktail strategy based on NK cell-derived exosomes and their biomimetic nanoparticles for dual tumor therapy. Cancers.

[133] Weng J., Xiang X., Ding L., Wong A.L.-A., Zeng Q., Sethi G., Wang L., Lee S.C., Goh B.C. (2021). Extracellular vesicles, the cornerstone of next-generation cancer diagnosis?. Semin. Cancer Biol..

[134] Jong A.Y., Wu C.-H., Li J., Sun J., Fabbri M., Wayne A.S., Seeger R.C. (2017). Large-scale isolation and cytotoxicity of extracellular vesicles derived from activated human natural killer cells. J. Extracell. Vesicles.

[135] Zhang M., Shao W., Yang T., Liu H., Guo S., Zhao D., Weng Y., Liang X.-J., Huang Y. (2022). Conscription of immune cells by light-activatable silencing NK-derived exosome (LASNEO) for synergetic tumor eradication. Adv. Sci..

